# Dataset of digital literacy of university students in Indonesia

**DOI:** 10.1016/j.dib.2024.111227

**Published:** 2024-12-16

**Authors:** Ermida Simanjuntak, Happy Cahaya Mulya, Agustina Engry, Ilham Nur Alfian

**Affiliations:** a Faculty of Psychology, Universitas Katolik Widya Mandala Surabaya, Raya Kalisari Selatan 1, Pakuwon City, Surabaya 60112, East Java, Indonesia; b Faculty of Psychology, Universitas Airlangga, Airlangga 4 - 6, Surabaya 60286, East Java, Indonesia

**Keywords:** Digital literacy, Internet use, University students, Indonesia

## Abstract

Digital literacy entails the ability to collect information, comprehend text on multimedia platforms, critically evaluate information, and communicate that information collaboratively. These skills are essential for university students to interact appropriately in cyberspace. This dataset aims to provide a comprehensive overview of digital literacy skills among university students in Indonesia. It comprises responses from 2921 university students (805 males and 2116 females) from various regions across the country. Data were measured using a digital literacy scale consisting of six subscales: technological skill, personal security skill, critical skill, device security skill, informational skill, and communication skill. This dataset plays an important role in describing the prevalent digital literacy competencies among Indonesian university students. It can contribute valuable information for the development of digital literacy programs to enhance these skills among Indonesian university students. Furthermore, this dataset holds academic significance for researchers interested in conducting cross-cultural comparisons of digital literacy trends among university students in different global contexts.

Specifications Table

**Table:** 

Subject	Social sciences
Specific subject area	Psychology
Type of data	Tables
Data collection	Data were collected using an online survey platform accessible via the link: bit.ly/literasidigitalUKWMS
Data source location	The data collection process involved the utilization of an online survey platform. The data collection was conducted in Indonesia.
Data accessibility	Data can be accessed on the Open Science Framework (OSF) via the following link: https://osf.io/2wndq/ with DOI 10.17605/OSF.IO/2WNDQ

## 1 Value of the Data

• This data describes digital literacy among university students in Indonesia, with a significant number of respondents successfully gathered from various regions in Indonesia: Sumatra, Java, Kalimantan, Bali, Sulawesi, Nusa Tenggara, and Maluku-Papua, representing 135 cities across the country.
• The students involved in providing this data come from various fields of study (67 fields), thus providing a diverse overview of digital literacy among Indonesian university students.
• This data will assist other researchers in mapping out the specific digital literacy skills of students, including technological skills, personal security skills, critical skills, device security skills, informational skills, and communication skills.
• This data serves as valuable information for designing appropriate training programs for the development of digital literacy among university students in Indonesia.
• This data will be beneficial for comparative analysis of digital literacy skills among students in other countries. Researchers interested in cross-cultural digital literacy studies can utilize this dataset.

## 2 Background

The development of internet use in Indonesia is increasing, providing wider opportunities for people to share information. However, the increasingly widespread use of the internet is not accompanied by the ability to use it wisely in society. A survey conducted by APJII in 2022 showed that 19–34 years old is one of the most significant internet users, which is 98.64 % of the total internet users in Indonesia [1]. Students belong to this category and this is also due to the learning process of students who use internet technology to find learning references [2]. On the other hand, students are often unable to sort out information, so they are vulnerable to becoming victims of hoaxes in cyberspace [3]. Referring to this condition, students are the ones who must get an education to be wise when interacting in cyberspace.

Several studies mention that digital literacy is one of the factors that can make a person respond wisely to various information in cyberspace [4]. Digital literacy is the ability to gather information, read and understand multimedia text, critically evaluate information, and communicate that information collaboratively [5]. Digital literacy skills are essential for students to interact well in cyberspace. The dataset will describe the digital literacy skills of university students in Indonesia.

## 3 Experimental Design, Materials and Methods

### 3.1 Participants and procedure

The participants in this study consist of undergraduate students from 135 cities across Indonesia. Respondents are currently enrolled in both public and private universities. The data were collected from 23 May until 10 June 2023. A total of 3015 respondents answered the questionnaire, although not all participants proceeded to complete the survey instrument. The respondents may decline participation so that they will not be directed to the questionnaire if they refuse to continue the participation. Consequently, 94 respondents were excluded from the analysis due to either refusal (45 respondents) or incomplete data (49 respondents). Subsequently, the dataset for analysis comprised responses from 2921 university students (805 males and 2116 females).

### 3.2 Materials and Methods

The survey instrument used in this research is derived from the digital literacy scale developed by Rodríguez-de-Dios et al. (2016) [6]. The scale comprises 28 items and assesses six dimensions of digital literacy: technological skills, personal security skills, critical skills, device security skills, informational skills, and communication skills. Respondents are required to respond to 28 statements (e.g. “I know how to bookmark a website I like so I can view it later” and “I know when I can post pictures and videos of other people online”). Each statement provides five response options, ranging from “Strongly Agree,” “Agree,” “Neutral,” “Disagree,” to “Strongly Disagree.” There are both favourable and unfavourable items in the digital literacy scale (see Table 1). The coding for favourable items is as follows: Strongly Agree = 5, Agree = 4, Neutral = 3, Disagree = 2, and Strongly Disagree = 1. This coding is reversed for unfavourable items. In the repository data, the coding has been conducted based on this standard. To ensure linguistic and conceptual equivalence, the scale by Rodríguez-de-Dios et al. (2016) [6] was translated and back-translated into Bahasa Indonesia by two proficient reviewers who are fluent in both English and Indonesian.

**Table 1:** The items of the digital literacy scale (Rodríguez-de-Dios et al., 2016) [6].

*Technological skill (TS)*
1. I know how to bookmark a website I like so I can view it later
2. I always know how to download/save a photo I found online
3. I know how to download information I found online
4. I always know how to connect to a Wi-Fi network, no matter the device or where I am
5. I know how to use shortcut keys (e.g., CTRL+C o cmd+C for copy)
6. I do not like downloading apps for smartphones as I find difficult to learn how to use them *(unfavourable)*
7. If I want to install new programs on my computer, I will ask someone to do it for me because I do not know *(unfavourable)*
*Personal security skill (PS)*
8. I know how to deactivate the function showing my geographical position (e.g., Facebook, apps)
9. I know when I can post pictures and videos of other people online
10. I know how to use 'report abuse' buttons on social media sites (e.g., someone uses my photo without my permission)
11. I know how to change the sharing settings of social media to choose what others can see about me (friends of friends, friends only, only me)
*Critical skill (CS)*
12. I know how to compare different sources to decide if information is true
13. I know how to determine if the information I find online is reliable
14. I know how to identify the author of the information and evaluate their reliability
15. I know how to compare different apps in order to choose which one is most reliable and secure
16. If I meet someone online, I know how to check if their profile is real
*Device security skill (DS)*
17. I use software to detect and remove viruses
18. I know how to detect a virus in my digital device
19.I know how to block unwanted or junk mail/spam
20. If something doesn't work occurs while I am using a device (computer, smartphone, etc.), I usually know what it is and how to fix the problem
*Informational skill (IS)*
21. I find hard to decide what the best keywords are for online searching *(unfavourable)*
22. I find confusing the way in which many websites are designed *(unfavourable)*
23. Sometimes I find difficult to determine how useful the information is for my purpose *(unfavourable)*
24. I get tired when looking for information online *(unfavourable)*
25. Sometimes I end up on websites without knowing how I got there *(unfavourable)*
*Communication skill (COS)*
26. Depending on who I want to communicate with, it is better to use one method over the other (make a call, send a WhatsApp message, send an email, etc.)
27. I know how to send any file to a contact using a smartphone
28. No matter with who I communicate: emojis are always useful *(unfavourable)*

## 4 Data Description

Data were collected from university students from various cities in Indonesia using the online survey platform bit.ly/literasidigitalUKWMS. The digital literacy questionnaire is written in the Indonesian language to ensure a comprehensive understanding of the questionnaire items by each respondent. It is also to facilitate respondents in answering according to their current digital literacy abilities. Sample collection was conducted by contacting key persons at each university and disseminating information about the survey along with the survey link through social media platforms such as Whatsapp, LINE, Instagram, and X (Twitter). There are 3015 university students responded to the online questionnaires and 45 students declined to participate by selecting “not agree to participate.” Additionally, 49 respondents did not complete the questionnaire, leaving some demographic questions, such as age and gender and providing incomplete responses to the digital literacy scales (not responding to more than 5 items on the digital literacy scale). Therefore, 2921 respondents were included in the final analysis, resulting in a usable data rate of 96.9 %.

The data were analyzed using SPSS and can be accessed at https://osf.io/2wndq/ with DOI 10.17605/OSF.IO/2WNDQ. There are 805 males (27.6 %) and 2116 females (72.4 %) with an age range between 18 and 25 years old (M = 19.15, SD = 0.95). The distribution of respondents by year of study is as follows: 1st year students: 19 respondents (0.65 %), 2nd year students: 2,608 respondents (89.28 %), 3rd year students: 105 respondents (3.59 %), 4th year students: 114 respondents (3.90 %), and students above the 4th year: 75 respondents (2.60 %). The respondents come from various regions in Indonesia: Sumatra (67 respondents), Java (2751 respondents), Kalimantan (27 respondents), Bali (24 respondents), Sulawesi (25 respondents), Nusa Tenggara (22 respondents), and Maluku-Papua (5 respondents). The highest number of respondents is from Java. This is because there are more higher education institutions in Java compared to other regions.

There are three questions about students' experience in online communities. These questions were developed for this data collection. These questions are intended to provide an understanding of the respondents' awareness of the accuracy of online information and their behavior in sharing information online. The questions are: “Do you know how to check hoaxes?”, “Do you check information before resharing?”, and “Would you reshare on social media if you were in an unpleasant condition?”. The respondents will answer “Yes” or “No” to these questions. For the first question, “Do you know how to check hoaxes?”, the majority of respondents (91.8 %) understand how to check hoaxes (2681 respondents), while only 8.2 % (240 respondents) do not understand how to check hoaxes on online information. When it comes to sharing information online, 98.1 % of respondents (2865 respondents) believe that they would check the information before resharing and 1.9 % of respondents (56 respondents) would not check the information before resharing. For the third question, “Would you reshare on social media if you were in an unpleasant condition?”, the majority of respondents, 90 % (2628 respondents) would not share their unpleasant condition on social media. Only 9.6 % (280 respondents) would share their unpleasant experiences, and 0.4 % (13 respondents) did not provide an answer (N/A).

Table 1 represents digital literacy scale items with six subscales: technological skill, personal security skill, critical skill, device security skill, informational skill, and communication skill. Composite variables were created by summing item scores within each subscale.

The factor loading values for the digital literacy scale items based on the first confirmatory factor analysis (CFA) are presented in Table 2. The first CFA test showed that the factor loading value for item 28 was negative, at -0.138, resulting in the exclusion of this item. Table 3 shows the factor loading values for the digital literacy scale after item 28 was excluded. The goodness of fit values for the digital literacy scale (after excluding item 28) are χ² = 3925; p = 0.00; RMSEA = 0.063; SRMR = 0.060; CFI = 0.878. RMSEA < 0.08 and SRMR < 0.10 meet the criteria for fit values [7].

**Table 2:** Factor loadings digital literacy items.

Factors	Items	Factor Loadings
Technological Skill (TS)	TS1	0.594
	TS2	0.730
	TS3	0.761
	TS4	0.560
	TS5	0.580
	TS6	0.377
	TS7	0.357
Personal Security Skill (PS)	PS8	0.638
	PS9	0.621
	PS10	0.742
	PS11	0.749
Critical Skill (CS)	CS12	0.802
	CS13	0.821
	CS14	0.692
	CS15	0.747
	CS16	0.560
Device Security Skill (DS)	DS17	0.702
	DS18	0.777
	DS19	0.605
	DS20	0.579
Informational Skill (IS)	IS21	0.616
	IS22	0.605
	ISL23	0.723
	IS24	0.651
	IS25	0.598
Communication Skill (COS)	COS26	0.215
	COS27	0.923
	COS28*	-0.138*

⁎ Factor loading value for item 28 is negative.

**Table 3:** Final factor loadings for digital literacy items*.

Factors	Items	Factor Loadings
Technological Skill (TS)	TS1	0.594
	TS2	0.730
	TS3	0.761
	TS4	0.560
	TS5	0.580
	TS6	0.377
	TS7	0.357
Personal Security Skill (PS)	PS8	0.638
	PS9	0.621
	PS10	0.742
	PS11	0.749
Critical Skill (CS)	CS12	0.802
	CS13	0.821
	CS14	0.692
	CS15	0.747
	CS16	0.560
Device Security Skill (DS)	DS17	0.702
	DS18	0.777
	DS19	0.605
	DS20	0.579
Informational Skill (IS)	IS21	0.616
	IS22	0.605
	ISL23	0.723
	IS24	0.651
	IS25	0.598
Communication Skill (COS)*	COS26	0.207
	COS27	0.958

⁎ Exclude item COS28.

Table 3 shows the factor loading values for the digital literacy scale after item 28 was excluded. The goodness of fit values for the digital literacy scale (after excluding item 28) are χ² = 3925; p = 0.00; RMSEA = 0.063; SRMR = 0.060; CFI = 0.878. RMSEA < 0.08 and SRMR < 0.10 meet the criteria for fit values [7]. The Cronbach's Alpha (α) coefficient of the final version of this scale is 0.895. Additionally, the Cronbach's Alpha coefficients for the six subscales are as follows: technological skill (α = 0.740), personal security skill (α = 0.769), critical skill (α = 0.836), device security skill (α = 0.753), informational skill (α = 0.773), and communication skill (α = 0.313).

Table 4 provides information about respondents' scores in specific aspects of digital literacy and overall digital literacy skills. Due to the varying number of items in each aspect, the scores listed under the digital literacy aspects are the average of the scores selected by the respondents for the digital literacy items. The overall digital literacy skill score is reported as the average of the total sum of all items on the digital literacy scale, excluding item number 28.

**Table 4:** Digital literacy scores of respondents (N = 2921).

Variables	M	SD	Minimum (Observed)	Maximum (Observed)	Minimum	Maximum
Technological skill	4.15	0.54	2.14	5	1	5
Personal security skill	4.37	0.57	1	5	1	5
Critical skill	4.02	0.62	2	5	1	5
Device security skill	3.71	0.71	1	5	1	5
Informational skill	3.26	0.76	1	5	1	5
Communication skill*	4.04	0.64	1	5	1	5
Digital literacy skill*	105.83	12.31	69	135	27	135

⁎ Exclude item number 28.

## Limitations

A limitation of this study is the self-reported nature of the respondents' assessments, which may lead to an overestimation of their digital literacy skills compared to their actual abilities. Another limitation is the relatively low reliability score for the communication skills aspect compared to the reliability scores of other digital literacy skill aspects. Additionally, the number of participants varies widely across different regions, with a higher concentration from the Java region compared to other regions in Indonesia. Therefore, it is necessary to have more participants from each region to better represent the digital literacy skills of Indonesian students. Moreover, the higher number of female respondents compared to male respondents could introduce a potential bias in the survey results. However, this research provides a brief overview of the digital literacy competence among university students in Indonesia.

## Ethics Statement

This research was approved by the Health Research Ethics Committee of the Faculty of Medicine, Universitas Katolik Widya Mandala Surabaya, with reference number: 0016/WM12/KEPK/DSN/T/2023.

## Credit Author Statement

**Ermida Simanjuntak:** conceptualization, methodology, data analysis, manuscript writing; **Happy Cahaya Mulya**: methodology, data gathering, data analysis, manuscript finalization, ethics process; **Agustina Engry**: methodology, data gathering, data analysis; **Ilham Nur Alfian**: data collection and methodology.

## Data Availability

Digital Literacy Data of Indonesian University Students (Original data).

## Appendix Supplementary materials

Image, application 1

Image, application 2

Image, application 3
