# Supplementary material for: Dataset of digital literacy of university students in Indonesia
Source: Data Brief. 2024 Dec 16;58:111227. doi: 10.1016/j.dib.2024.111227 (PMC11729011; doi:10.1016/j.dib.2024.111227)
Supplement: Supplementary file 1 [file mmc1.pdf]

DIGITAL LITERACY SURVEY CODE BOOK

1. Where is your hometown? (name of the city)
   All data will be coded using the names of the cities of the respondents.

2. What is your gender?
   All data will be coded based on the gender chosen by the respondents (Male/Female).

3. How old are you? (write the number)
   All data will be coded based on the numbers (age) provided by the respondents.

4. What is your field of study?
   All data will be coded based on the name of the city provided by the respondents.

5. In which academic year did you start your study in the university? (write the year)
   All data will be coded based on the numbers (academic year of study) provided by the respondents.

6. Do you know how to check hoaxes?
   All data will be coded based on the answer chosen by the respondents (Yes/No).

7. Do you check the information first before resharing?
   All data will be coded based on the answer chosen by the respondents (Yes/No).

8. Would you reshare in social media if you were in an unpleasant condition?
   All data will be coded based on the answer chosen by the respondents (Yes/No).

INSTRUCTIONS

The following statements concern about your internet usage history.
Please mark your response by clicking to one of the response options below :

- ☐ Strongly Agree
- ☐ Agree
- ☐ Neutral
- ☐ Disagree
- ☐ Strongly Disagree

Please respond based on your actual experiences.

For Item Favourable will be coded as below :

Strongly Agree	= 5
Agree	= 4
Neutral	= 3
Disagree	= 2
Strongly Disagree	= 1

For Item Unfavourable will be coded as below :

Strongly Agree	= 1
Agree	= 2
Neutral	= 3
Disagree	= 4
Strongly Disagree	= 5

1. I know how to bookmark a website I like so I can view it later. **(FAVOURABLE)**

Strongly Agree	= 5
Agree	= 4
Neutral	= 3
Disagree	= 2
Strongly Disagree	= 1

2. I always know how to download/save a photo I found online. **(FAVOURABLE)**

Strongly Agree	= 5
Agree	= 4
Neutral	= 3
Disagree	= 2
Strongly Disagree	= 1

3. I know how to download information I found online. **(FAVOURABLE)**

Strongly Agree	= 5
Agree	= 4
Neutral	= 3
Disagree	= 2
Strongly Disagree	= 1

4. I always know how to connect to a Wi-Fi network, no matter the device or where I am. **(FAVOURABLE)**

Strongly Agree	= 5
Agree	= 4
Neutral	= 3
Disagree	= 2
Strongly Disagree	= 1

5. I know how to use shortcut keys (e.g., CTRL+C o cmd+C for copy). **(FAVOURABLE)**

Strongly Agree	= 5
Agree	= 4
Neutral	= 3
Disagree	= 2
Strongly Disagree	= 1

6. I do not like downloading apps for smartphones as I find difficult to learn how to use them. **(UNFAVOURABLE)**
   Strongly Agree = 1
   Agree = 2
   Neutral = 3
   Disagree = 4
   Strongly Disagree = 5

7. If I want to install new programs on my computer, I will ask someone to do it for me because I do not know. **(UNFAVOURABLE)**
   Strongly Agree = 1
   Agree = 2
   Neutral = 3
   Disagree = 4
   Strongly Disagree = 5

8. I know how to deactivate the function showing my geographical position (e.g., Facebook, apps). **(FAVOURABLE)**
   Strongly Agree = 5
   Agree = 4
   Neutral = 3
   Disagree = 2
   Strongly Disagree = 1

9. I know when I can post pictures and videos of other people online. **(FAVOURABLE)**
   Strongly Agree = 5
   Agree = 4
   Neutral = 3
   Disagree = 2
   Strongly Disagree = 1

10. I know how to use 'report abuse' buttons on social media sites (e.g., someone uses my photo without my permission). **(FAVOURABLE)**
    Strongly Agree = 5
    Agree = 4
    Neutral = 3
    Disagree = 2
    Strongly Disagree = 1

11. I know how to change the sharing settings of social media to choose what others can see about me (friends of friends, friends only, only me). **(FAVOURABLE)**
    Strongly Agree = 5
    Agree = 4
    Neutral = 3
    Disagree = 2
    Strongly Disagree = 1

12. I know how to compare different sources to decide if information is true. **(FAVOURABLE)**
    Strongly Agree = 5
    Agree = 4
    Neutral = 3
    Disagree = 2
    Strongly Disagree = 1

13. I know how to determine if the information I find online is reliable. **(FAVOURABLE)**
    Strongly Agree = 5
    Agree = 4
    Neutral = 3
    Disagree = 2
    Strongly Disagree = 1

14. I know how to identify the author of the information and evaluate their reliability. **(FAVOURABLE)**
    Strongly Agree = 5
    Agree = 4
    Neutral = 3
    Disagree = 2
    Strongly Disagree = 1

15. I know how to compare different apps in order to choose which one is most reliable and secure. **(FAVOURABLE)**
    Strongly Agree = 5
    Agree = 4
    Neutral = 3
    Disagree = 2
    Strongly Disagree = 1

16. If I meet someone online, I know how to check if their profile is real. **(FAVOURABLE)**
    Strongly Agree = 5
    Agree = 4
    Neutral = 3
    Disagree = 2
    Strongly Disagree = 1

17. I use software to detect and remove viruses. **(FAVOURABLE)**
    Strongly Agree = 5
    Agree = 4
    Neutral = 3
    Disagree = 2
    Strongly Disagree = 1

18. I know how to detect a virus in my digital device. **(FAVOURABLE)**
Strongly Agree = 5
Agree = 4
Neutral = 3
Disagree = 2
Strongly Disagree = 1

19. I know how to block unwanted or junk mail/spam. **(FAVOURABLE)**
Strongly Agree = 5
Agree = 4
Neutral = 3
Disagree = 2
Strongly Disagree = 1

20. If something doesn't work occurs while I am using a device (computer, smartphone, etc.), I usually know what it is and how to fix the problem. **(FAVOURABLE)**
Strongly Agree = 5
Agree = 4
Neutral = 3
Disagree = 2
Strongly Disagree = 1

21. I find hard to decide what the best keywords are for online searching. **(UNFAVOURABLE)**
Strongly Agree = 1
Agree = 2
Neutral = 3
Disagree = 4
Strongly Disagree = 5

22. I find confusing the way in which many websites are designed. **(UNFAVOURABLE)**
Strongly Agree = 1
Agree = 2
Neutral = 3
Disagree = 4
Strongly Disagree = 5

23. Sometimes I find difficult to determine how useful the information is for my purpose. **(UNFAVOURABLE)**
Strongly Agree = 1
Agree = 2
Neutral = 3
Disagree = 4
Strongly Disagree = 5

24. I get tired when looking for information online. **(UNFAVOURABLE)**
    Strongly Agree = 1
    Agree = 2
    Neutral = 3
    Disagree = 4
    Strongly Disagree = 5

25. Sometimes I end up on websites without knowing how I got there. **(UNFAVOURABLE)**
    Strongly Agree = 1
    Agree = 2
    Neutral = 3
    Disagree = 4
    Strongly Disagree = 5

26. Depending on who I want to communicate with, it is better to use one method over the other (make a call, send a WhatsApp message, send an email, etc.). **(FAVOURABLE)**
    Strongly Agree = 5
    Agree = 4
    Neutral = 3
    Disagree = 2
    Strongly Disagree = 1

27. I know how to send any file to a contact using a smartphone. **(FAVOURABLE)**
    Strongly Agree = 5
    Agree = 4
    Neutral = 3
    Disagree = 2
    Strongly Disagree = 1

28. No matter with who I communicate: emojis are always useful. **(UNFAVOURABLE)**
    Strongly Agree = 1
    Agree = 2
    Neutral = 3
    Disagree = 4
    Strongly Disagree = 5

The Digital Literacy Scale consists of 5 subscales, with items for each subscale as follows:

1. Technology Skill (TS): Items numbered 1 – 7
2. Personal Security Skill (PS): Items numbered 8 –11
3. Critical Skill (CS): Items numbered 12 – 16
4. Device Security Skill (DS): Items numbered 17 – 20
5. Informational Skill (IS): Items numbered 21 – 25
6. Communication Skill (COS): Items numbered 26 – 28
