# Supplementary material for: Dataset of digital literacy of university students in Indonesia
Source: Data Brief. 2024 Dec 16;58:111227. doi: 10.1016/j.dib.2024.111227 (PMC11729011; doi:10.1016/j.dib.2024.111227)
Supplement: Supplementary file 2 [file mmc2.pdf]

INFORMED CONSENT E-SURVEY

This research aims to describe the digital literacy among students in Indonesia. The questionnaire consists of three parts and takes approximately 20 minutes to complete.

If you are willing to participate in this research, you can click on the “YES” button at the end of this page.

Participation in this research is voluntary, so you can withdraw your consent at any time if you feel uncomfortable. If you have any questions regarding the survey and this research, you may contact us via email at **mida@ukwms.ac.id.**

Your participation is highly valuable for the development of research in the area of Cyber behaviour. We deeply appreciate your kind attention.

Research Team,
Ermida Simanjuntak (mida@ukwms.ac.id)
Happy Cahaya Mulia (happycahaya@ukwms.ac.id)
Agustina Engry (agustina-engry@ukwms.ac.id)
Ilham Nur Alfian (ilham.nuralfian@psikologi.unair.ac.id)

Participants Name : ___________________________________

Date : ___________________________________ (Month/Date/Year)

Are you willing to participate in this research?

☐ Yes
☐ No

If you click “Yes”, you will be directed to the survey page.
Thank you for your kind attention.
