# Supplementary material for: Dataset of digital literacy of university students in Indonesia
Source: Data Brief. 2024 Dec 16;58:111227. doi: 10.1016/j.dib.2024.111227 (PMC11729011; doi:10.1016/j.dib.2024.111227)
Supplement: Supplementary file 3 [file mmc3.pdf]

DIGITAL LITERACY SURVEY

1. Where is your hometown? (name of the city)

   ____

2. What is your gender?
   - ☐ Male
   - ☐ Female

3. How old are you? (write the number)

   ____

4. What is your field of study?

   ____

5. In which academic year did you start your study in the university? (write the year)

   ____

6. Do you know how to check hoaxes?
   - ☐ Yes
   - ☐ No

7. Do you check the information first before resharing?
   - ☐ Yes
   - ☐ No

8. Would you reshare in social media if you were in an unpleasant condition?
   - ☐ Yes
   - ☐ No

INSTRUCTIONS

The following statements concern about your internet usage history.
Please mark your response by clicking to one of the response options below :

- ☐ Strongly Agree
- ☐ Agree
- ☐ Neutral
- ☐ Disagree
- ☐ Strongly Disagree

Please respond based on your actual experiences.

1. I know how to bookmark a website I like so I can view it later.
   - ☐ Strongly Agree
   - ☐ Agree
   - ☐ Neutral
   - ☐ Disagree
   - ☐ Strongly Disagree

2. I always know how to download/save a photo I found online.
   - ☐ Strongly Agree
   - ☐ Agree
   - ☐ Neutral
   - ☐ Disagree
   - ☐ Strongly Disagree

3. I know how to download information I found online.
   - ☐ Strongly Agree
   - ☐ Agree
   - ☐ Neutral
   - ☐ Disagree
   - ☐ Strongly Disagree

4. I always know how to connect to a Wi-Fi network, no matter the device or where I am.
   - ☐ Strongly Agree
   - ☐ Agree
   - ☐ Neutral
   - ☐ Disagree
   - ☐ Strongly Disagree

5. I know how to use shortcut keys (e.g., CTRL+C o cmd+C for copy).
   - ☐ Strongly Agree
   - ☐ Agree
   - ☐ Neutral
   - ☐ Disagree
   - ☐ Strongly Disagree

6. I do not like downloading apps for smartphones as I find difficult to learn how to use them.
   - ☐ Strongly Agree
   - ☐ Agree
   - ☐ Neutral
   - ☐ Disagree
   - ☐ Strongly Disagree

7. If I want to install new programs on my computer, I will ask someone to do it for me because I do not know.
   - ☐ Strongly Agree
   - ☐ Agree
   - ☐ Neutral
   - ☐ Disagree
   - ☐ Strongly Disagree

8. I know how to deactivate the function showing my geographical position (e.g., Facebook, apps).
   - ☐ Strongly Agree
   - ☐ Agree
   - ☐ Neutral
   - ☐ Disagree
   - ☐ Strongly Disagree

9. I know when I can post pictures and videos of other people online.
   - ☐ Strongly Agree
   - ☐ Agree
   - ☐ Neutral
   - ☐ Disagree
   - ☐ Strongly Disagree

10. I know how to use 'report abuse' buttons on social media sites (e.g., someone uses my photo without my permission).
   - ☐ Strongly Agree
   - ☐ Agree
   - ☐ Neutral
   - ☐ Disagree
   - ☐ Strongly Disagree

11. I know how to change the sharing settings of social media to choose what others can see about me (friends of friends, friends only, only me).
   - ☐ Strongly Agree
   - ☐ Agree
   - ☐ Neutral
   - ☐ Disagree
   - ☐ Strongly Disagree

12. I know how to compare different sources to decide if information is true.
   - ☐ Strongly Agree
   - ☐ Agree
   - ☐ Neutral
   - ☐ Disagree
   - ☐ Strongly Disagree

13. I know how to determine if the information I find online is reliable.
   - ☐ Strongly Agree
   - ☐ Agree
   - ☐ Neutral
   - ☐ Disagree
   - ☐ Strongly Disagree

14. I know how to identify the author of the information and evaluate their reliability.
    - ☐ Strongly Agree
    - ☐ Agree
    - ☐ Neutral
    - ☐ Disagree
    - ☐ Strongly Disagree

15. I know how to compare different apps in order to choose which one is most reliable and secure.
    - ☐ Strongly Agree
    - ☐ Agree
    - ☐ Neutral
    - ☐ Disagree
    - ☐ Strongly Disagree

16. If I meet someone online, I know how to check if their profile is real.
    - ☐ Strongly Agree
    - ☐ Agree
    - ☐ Neutral
    - ☐ Disagree
    - ☐ Strongly Disagree

17. I use software to detect and remove viruses.
    - ☐ Strongly Agree
    - ☐ Agree
    - ☐ Neutral
    - ☐ Disagree
    - ☐ Strongly Disagree

18. I know how to detect a virus in my digital device.
    - ☐ Strongly Agree
    - ☐ Agree
    - ☐ Neutral
    - ☐ Disagree
    - ☐ Strongly Disagree

19. I know how to block unwanted or junk mail/spam.
    - ☐ Strongly Agree
    - ☐ Agree
    - ☐ Neutral
    - ☐ Disagree
    - ☐ Strongly Disagree

20. If something doesn't work occurs while I am using a device (computer, smartphone, etc.), I usually know what it is and how to fix the problem.
    ☐ Strongly Agree
    ☐ Agree
    ☐ Neutral
    ☐ Disagree
    ☐ Strongly Disagree

21. I find hard to decide what the best keywords are for online searching.
    ☐ Strongly Agree
    ☐ Agree
    ☐ Neutral
    ☐ Disagree
    ☐ Strongly Disagree

22. I find confusing the way in which many websites are designed.
    ☐ Strongly Agree
    ☐ Agree
    ☐ Neutral
    ☐ Disagree
    ☐ Strongly Disagree

23. Sometimes I find difficult to determine how useful the information is for my purpose.
    ☐ Strongly Agree
    ☐ Agree
    ☐ Neutral
    ☐ Disagree
    ☐ Strongly Disagree

24. I get tired when looking for information online.
    ☐ Strongly Agree
    ☐ Agree
    ☐ Neutral
    ☐ Disagree
    ☐ Strongly Disagree

25. Sometimes I end up on websites without knowing how I got there.
    ☐ Strongly Agree
    ☐ Agree
    ☐ Neutral
    ☐ Disagree
    ☐ Strongly Disagree

26. Depending on who I want to communicate with, it is better to use one method over the other (make a call, send a WhatsApp message, send an email, etc.).
    - ☐ Strongly Agree
    - ☐ Agree
    - ☐ Neutral
    - ☐ Disagree
    - ☐ Strongly Disagree

27. I know how to send any file to a contact using a smartphone.
    - ☐ Strongly Agree
    - ☐ Agree
    - ☐ Neutral
    - ☐ Disagree
    - ☐ Strongly Disagree

28. No matter with who I communicate: emojis are always useful.
    - ☐ Strongly Agree
    - ☐ Agree
    - ☐ Neutral
    - ☐ Disagree
    - ☐ Strongly Disagree

Thank you for your participation and kindly check to ensure that no numbers are missed.
